# Regulatory T cells characterized by low Id3 expression are highly suppressive and accumulate during chronic infection

**DOI:** 10.18632/oncotarget.22159

**Published:** 2017-10-27

**Authors:** Katharina S. Rauch, Miriam Hils, Alexandra J. Menner, Mikael Sigvardsson, Susana Minguet, Peter Aichele, Christian Schachtrup, Kristina Schachtrup

**Affiliations:** ^1^ Center for Chronic Immunodeficiency (CCI), Medical Center – University of Freiburg, Faculty of Medicine, Freiburg, Germany; ^2^ Faculty of Biology, Freiburg, Germany; ^3^ Institution for Clinical and Experimental Sciences, Faculty of Health Sciences, Linköping University, Linköping, Sweden; ^4^ Centre for Biological Signalling Studies BIOSS, University of Freiburg, Germany and Max Planck Institute of Immunology and Epigenetics, Freiburg, Germany; ^5^ Institute for Immunology, Medical Center, University of Freiburg, Faculty of Medicine, Freiburg, Germany; ^6^ Institute of Anatomy and Cell Biology, Faculty of Medicine, Freiburg, Germany

**Keywords:** Treg cells, chronic infection, transcription, Id proteins, immunology

## Abstract

Foxp3^+^ regulatory T (Treg) cells are broadly divided into naive-like and activated Treg cells, however recent studies suggest further Treg cell heterogeneity. Treg cells contribute to impaired T cell responses in chronic infections, but the role of specific Treg cell subpopulations in viral infections is not well defined. Here, we report that activated Treg cells are separated into two transcriptionally distinct subpopulations characterized by low or high expression of the transcriptional regulator *Id3*. *Id3*^lo^ Treg cells are a highly suppressive Treg cell subpopulation, expressing elevated levels of immunomodulatory molecules and are capable of broadly targeting T cell responses. Viral infection and interleukin-2 promote the differentiation of *Id3*^hi^ into *Id3*^lo^ Treg cells and during chronic infection *Id3*^lo^ Treg cells are the predominant Treg cell population. Thus, our report provides a framework, in which different activated Treg cell subpopulations specifically affect immune responses, possibly contributing to T cell dysfunction in chronic infections.

## INTRODUCTION

CD4^+^Foxp3^+^ regulatory T (Treg) cells are crucial for maintaining immune homeostasis and self-tolerance due to their ability to suppress T cell responses. Consequently, mice and humans lacking Treg cells suffer from autoimmune and inflammatory disorders [1, 2]. Besides their well-established role in suppressing autoreactive T cells, Treg cells also fulfill diverse roles promoting or preventing effective immune responses upon infection [3]. Treg cell abundance is tightly balanced for optimal pathogen-specific responses [3]. Treg cell numbers are transiently decreased in many acute infections to allow an optimal immune response and increased Treg cell numbers or activity can impair effector T cell responses resulting in pathogen persistence. Thus, in acute lymphocytic choriomeningitis virus (LCMV) infection, a failure to reduce Treg cell abundance impairs anti-viral T cell responses [4] and in persistent LCMV infection, increased Treg cell numbers contribute to dysregulated T cell responses, which are restored upon Treg cell depletion [5–7]. Most interestingly however, the appropriate magnitude and quality of the Treg cell response can be beneficial for the host by allowing the localization of effector immune cells into lymph nodes and infected tissues [8] and by supporting memory T cell formation [9–11].

Treg cells can be broadly divided into naive-like/central CD44^lo^CD62L^hi^ Treg cells and activated CD44^hi^CD62L^lo/-^ Treg cells [12, 13]. Treg cells leaving the thymus belong to the naive-like Treg cell population, characterized by quiescence, expression of high levels of antiapoptotic molecules and recirculation through secondary lymphoid tissues. Treg cells differentiate into more suppressive CD44^hi^ Treg cells upon T cell receptor-mediated activation in the presence of inflammatory signals [12]. Activated Treg cells are further classified based on their phenotypic and functional diversity. This specialization is controlled by the inflammatory milieu wherein they are activated, similarly to the polarization of CD4^+^ T helper (Th) cells into Th1, Th2, Th17 and other Th cell subsets [14, 15]. These specialized Treg cells co-differentiate with their related Th cell subsets and are specifically tailored to suppress their respective Th cell subset [14]. Treg cell specialization is regulated by the same transcription factors that also control the differentiation of each specific Th cell subset. In line with this, Treg cells expressing the Th1 specific transcription factor T-bet are essential for suppression of Th1 responses [16] and Treg cells deficient in the Th17 associated transcription factor STAT3 selectively fail to suppress Th17 responses [17]. Treg cells are also heterogeneous with regard to the effector mechanisms they employ to suppress immune responses. These include the production of secreted factors such as IL10, TGFβ, IL35, Granzyme B, fibrinogen-like protein-2 (FGL2) and the up-regulation of inhibitory receptors, such as CTLA4, LAG3 or PD1 and others [14, 15]. The expression of these effector molecules is controlled by specific sets of transcriptional regulators. For example, BLIMP1 is essential for *Il10* expression and ICOS up-regulation in Treg cells [18] and ligation of TIGIT on Treg cells results in the up-regulation of C/EBPα and the expression of *Fgl2* [19].

Id proteins (Id1-4), especially Id2 and Id3, are major regulators of immune cell development and specialization [20]. Id proteins are transcriptional regulators controlling the activity of the basic helix-loop-helix transcription factors of the E protein family through heterodimerization and subsequent prevention of DNA binding by the E proteins [21, 22]. Specifically Id3 controls T cell differentiation at various stages of development and specification [23–28]. In Treg cells, Id3 maintains Foxp3 expression [29] and together with its related factor Id2 it guarantees Treg cell survival and controls Treg cell homing [28]. Yet, whether Id3 regulates Treg cell specification during immune responses is not known.

In this study, we show that differential expression of the transcriptional regulator Id3 allows the separation of activated CD44^hi^ Treg cells into two transcriptionally and functionally distinct subpopulations. CD44^hi^ Treg cells expressing high *Id3* levels (*Id3*^hi^) showed a naive-like Treg cell transcriptional profile. In contrast, CD44^hi^ Treg cells with low or absent Id3 expression (*Id3*^lo^) transcriptionally and phenotypically resembled effector Treg cells. CD44^hi^*Id3*^lo^ Treg cells were superior in suppressing Th cell differentiation. Acute LCMV infection promoted the differentiation of *Id3*^hi^ into *Id3*^lo^ Treg cells and persistent LCMV infection preferentially increased the abundance of the *Id3*^lo^ Treg cell subtype. These findings support a specific role of *Id3*^lo^ Treg cells in contributing to the manifestation of T cell dysfunction during chronic viral infection.

## RESULTS

### Id3 expression separates Treg cells in two transcriptionally distinct populations

**Figure 1:**
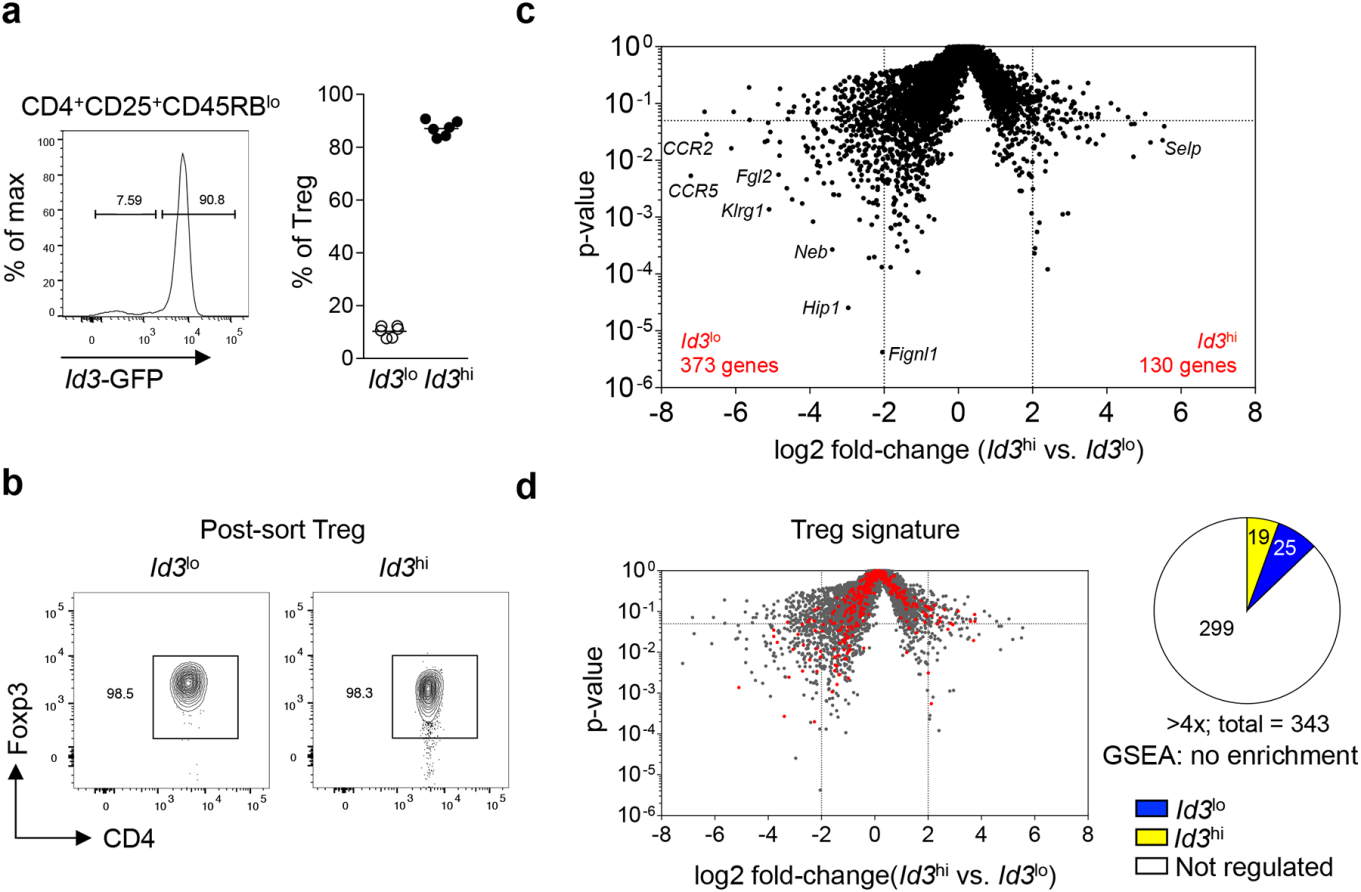
*Id3* expression separates Treg cells into two a transcriptionally distinct populations **(a)** *Id3* expression in splenic Treg cells (CD4^+^CD25^+^CD45RB^lo^) from naive *Id3*^GFP/+^ mice using flow cytometry (left) and quantification of the percentage of *Id3*^lo^ and *Id3*^hi^ cells among splenic Treg cells (right). Each symbol represents an individual mouse and horizontal lines are the mean. **(b)** Foxp3 expression of sorted *Id3*^lo^ and *Id3*^hi^ splenic Treg cells (CD4^+^CD25^+^CD45RB^lo^) from naive *Id3*^GFP/+^ mice by flow cytometry. **(c)** Volcano plot comparing the p-value versus log2 fold-change in gene expression of *Id3*^hi^ versus *Id3*^lo^ Treg cells. **(d)** Volcano plot from (c) with canonical Treg cell signature genes [30] highlighted in red. Pie chart shows the number of Treg cell signature genes up-regulated more than 4-fold in *Id3*^lo^ (blue) or *Id3*^hi^ (yellow) Treg cells.

Although the majority of Treg cells express high levels of *Id3*, recent data suggests the presence of an *Id3*^lo^ Treg cell population [28]. To detect *Id3* expression in Treg cells on a single cell level, we used *Id3*-GFP reporter mice (*Id3*^GFP/+^), expressing GFP under the control of the endogenous *Id3* locus [24]. As shown before, about 90% of Treg cells (CD4^+^CD25^+^CD45RB^lo^) in spleen and lymph nodes showed high *Id3* expression and around 10% of Treg cells had low to absent *Id3* expression under homeostatic conditions (Figure 1a) [28]. To investigate if *Id3* expression levels separate Treg cells into distinct subpopulations, we determined the transcriptional profile of sorted *Id3*^lo^ and *Id3*^hi^ Treg cells from naive *Id3*^GFP/+^ mice. Equal Foxp3 protein expression in *Id3*^lo^ and *Id3*^hi^ CD4^+^CD25^+^CD45RB^lo^ cells confirmed that both populations were indeed Foxp3 expressing Treg cells (Figure 1b). 373 genes with a greater than 4-fold increased expression in *Id3*^lo^ compared to *Id3*^hi^ Treg cells and 130 genes with greater than 4-fold increased expression in *Id3*^hi^ compared to *Id3*^lo^ Treg cells were identified by RNA sequencing analysis (Figure 1c). Gene set enrichment analysis (GSEA) of the transcriptional profiles of *Id3*^lo^ and *Id3*^hi^ Treg cells with the Treg cell transcriptional signature [30] showed no differences in the expression of Treg cell signature genes (Figure 1d), confirming that both subpopulations belong to the Treg cell lineage. Yet, their substantial difference in gene expression suggests that *Id3*^lo^ and *Id3*^hi^ Treg cells are two distinct Treg cell subpopulations.

### The transcriptional profile of *Id3*^lo^ Treg cells indicates a highly activated phenotype

**Figure 2:**
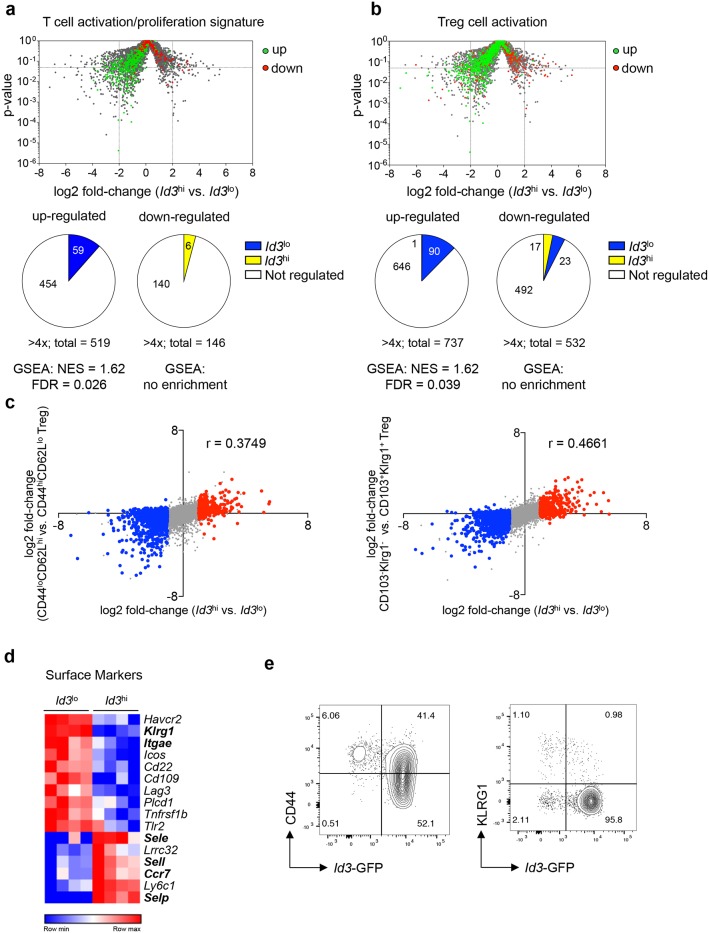
*Id3*^lo^ Treg cells express a transcriptional profile resembling activated Treg cells **(a, b)** Volcano plot from Figure 1c superimposed with genes up- (green) or down-regulated (red) in (a) T cell activation/proliferation transcriptional signatures [30] or (b) in Treg cell activation [31]. Pie charts show the number of genes up-regulated more than 4-fold in *Id3*^lo^ (blue) or *Id3*^hi^ (yellow) Treg cells. **(c)** Difference in transcript abundance in *Id3*^hi^ versus *Id3*^lo^ Treg cells plotted against that in CD44^lo^CD62L^hi^ versus CD44^hi^CD62L^lo^ Treg cells [33] (left), and plotted against that in CD103^-^KLRG1^-^ versus CD103^+^KLRG1^+^ Treg cells [32] (right). Colors indicate transcripts significantly up-regulated greater than 2-fold in *Id3*^lo^ (blue) or *Id3*^hi^ (red) Treg cells. The r-value of Spearman correlation is indicated. **(d)** Heatmap of genes encoding for surface markers that are differentially (> 4-fold) expressed in splenic *Id3*^hi^ compared to *Id3*^lo^ Treg cells (CD4^+^CD25^+^CD45RB^lo^) of naive *Id3*^GFP/+^ mice. RNA was obtained from 4 independent experiments. **(e)** *Id3* expression versus CD44 (left) or KLRG1 (right) expression in splenic Treg cells (CD4^+^CD25^+^) from naive *Id3*^GFP/+^ mice. Data are representative from at least 2 individual mice.

To gain insights into specific functional properties of *Id3*^lo^ and *Id3*^hi^ Treg cells, we performed GSEA with known transcriptional profiles of different T cell and Treg cell subpopulations. These analysis showed that *Id3*^lo^ Treg cells preferentially expressed genes matching the T cell activation and proliferation signature [18, 30] (Figure 2a). Additionally, *Id3*^lo^ Treg cells expressed genes that are up-regulated upon activation of Treg cells [31] (Figure 2b). Classically, activated Treg cells are separated from naive-like/central CD44^lo^CD62L^hi^ Treg cells by a CD44^hi^CD62L^lo/-^ surface phenotype [12]. Alternatively, CD103^+^KLRG1^+^ and TIGIT^+^ Treg cells are highly proliferative and suppressive Treg cell subsets [32, 19]. Comparison of gene expression data showed that the transcriptional signature of *Id3*^lo^ versus *Id3*^hi^ Treg cells correlated with: (1) changes in expression occurring during the transition of CD44^lo^CD62L^hi^ naive-like toCD44^hi^CD62L^lo/-^ Treg cells [33] (Figure 2c, left), (2) the transcriptional profile of CD103^+^KLRG1^+^ Treg cells [32] (Figure 2c, right) and (3) the transcriptional profile of TIGIT^+^ Treg cells [19], albeit to a lesser extend (Supplementary Figure 1a). As a control, there was no correlation with genes differentially expressed between Treg and CD4^+^ conventional T cells [30] (Supplementary Figure 1b). Analysis of differential expression of cell surface markers showed that *Id3*^lo^ Treg cells expressed high levels of KLRG1 and CD103 (*Itgae*) (Figure 2d), which are exclusively found in highly suppressive effector Treg cells [32]. In contrast, *Id3*^hi^ Treg cells preferentially expressed selectins E (*Sele*), L (*Sell*) and P (*Selp*) and the chemokine receptor CCR7, which are important for homing and predominantly expressed in naive-like Treg cells (Figure 2d). In summary, *Id3*^lo^ Treg cells expressed genes predominantly found in activated Treg cells.

To investigate, if all activated Treg cells were *Id3*^lo^, we determined the abundance of *Id3*^lo^ Treg cells among CD44^hi^ and KLRG1^+^ Treg cells. As expected from their transcriptional profile, all *Id3*^lo^ Treg cells expressed high levels of CD44 (Figure 2e), but although their transcriptional profile correlated with that of CD103^+^KLRG1^+^ Treg cells only one third of *Id3*^lo^ Treg cells were KLRG1^+^ (Figure 2e). *Id3*^hi^ Treg cells contained CD44^lo^ and CD44^hi^ Treg cells, but almost no KLRG1^+^ cells (Figure 2e). Thus, highly suppressive KLRG1^+^ cells predominantly express low *Id3* levels, while activated CD44^hi^ Treg cells are separate into two distinct cell populations based on the expression of *Id3*.

### *Id3*^lo^ Treg cells are highly suppressive effector Treg cells

**Figure 3:**
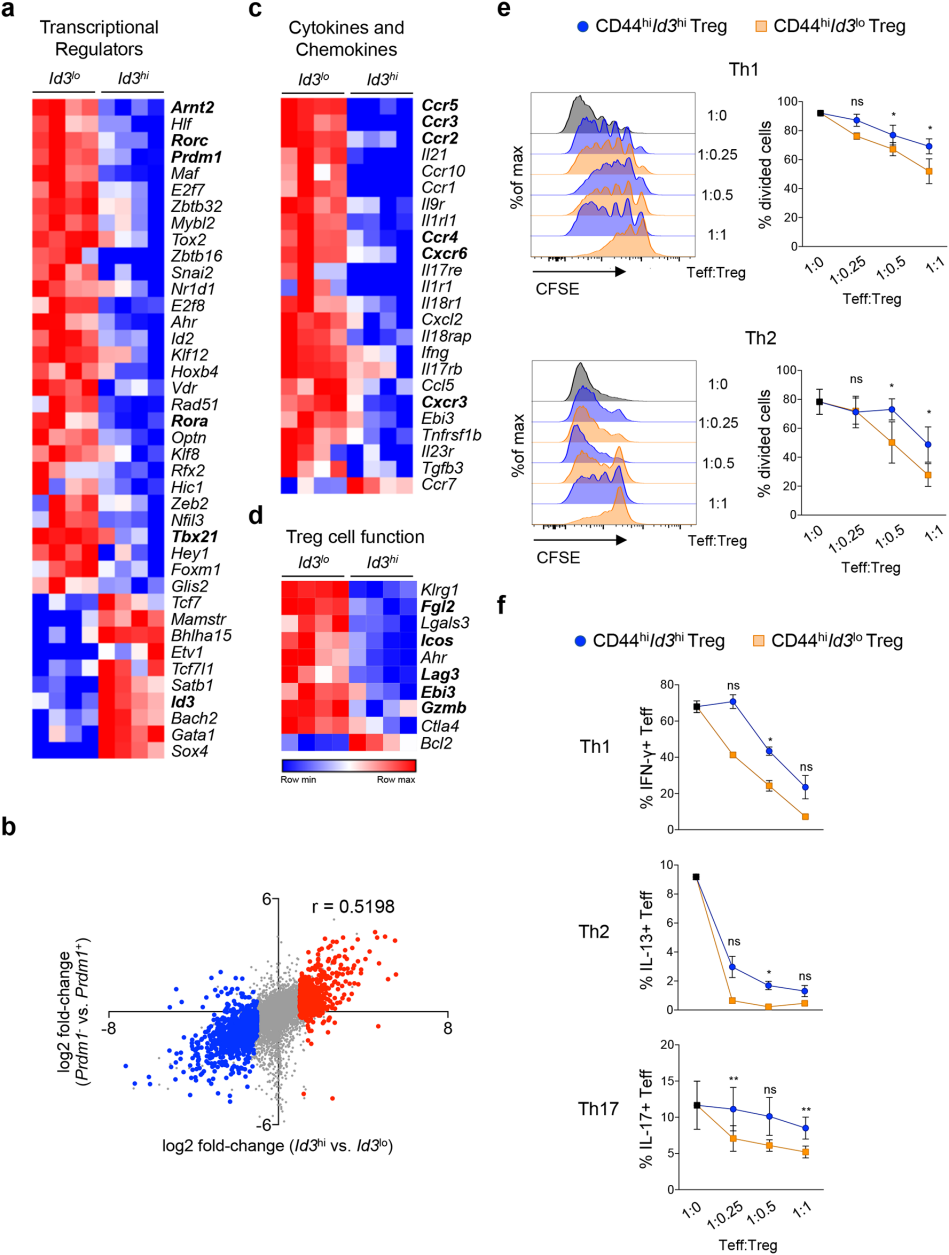
*Id3*^lo^ Treg cells are highly suppressive **(a)** Heatmap of genes encoding for transcriptional regulators that are differentially (> 4-fold) expressed in splenic *Id3*^hi^ compared to *Id3*^lo^ Treg cells (CD4^+^CD25^+^CD45RB^lo^) of naive *Id3*^GFP/+^ mice. RNA was obtained from 4 independent experiments. **(b)** Difference in transcript abundance in *Id3*^hi^ versus *Id3*^lo^ Treg cells plotted against that in *Prdm1*^-^ versus *Prdm1*^+^ Treg cells [35]. Colors indicate transcripts significantly up-regulated more than 2-fold in *Id3*^lo^ (blue) or *Id3*^hi^ (red) Treg cells. The r-value of Spearman correlation is indicated. **(c, d)** Heatmaps of genes encoding for (c) cytokines, chemokines and their receptors and (d) genes encoding for molecules involved in Treg cell function that are differentially (> 4-fold) expressed in splenic *Id3*^hi^ compared to *Id3*^lo^ Treg cells (CD4^+^CD25^+^CD45RB^lo^) of naive *Id3*^GFP/+^ mice. RNA was obtained from 4 independent isolations. **(e, f)** 10^5^ CFSE labeled CD4^+^ effector T (Teff) cells (Thy1.1) were co-cultured under Th1, Th2 or Th17 cell polarizing conditions with sorted CD44^hi^*Id3*^hi^ (blue) or CD44^hi^*Id3*^lo^ (orange) Treg cells (CD4^+^CD25^hi^) from IL2/IL2mAb treated *Id3*^GFP/+^ mice (Thy1.2). (e) Representative CFSE fluorescence of Teff cells (Thy1.1^+^) cultured at the indicated Treg/Teff cell ratios and quantification of the percentage of divided Teff cells. Mean ± SEM from 3 independent experiments are shown. ^*^p < 0.05; ns = not significant (paired Student’s t test). (f) Flow cytometric quantification of the percentage of Teff cells (Thy1.1^+^) expressing IFNγ, IL13 or IL17A after restimulation with PMA/Iono for 4 hours. Mean ± SEM from 3 independent experiments (Th1, Th17) or mean ± SEM from several wells of 1 representative experiment (Th2) are shown. ^*^p < 0.05; ^**^p < 0.01; ns = not significant (Student’s t test).

Specialized Treg cell subpopulations tailored to suppress Th1, Th2, Th17 or follicular helper T (Tfh) cell responses are defined by the expression of specific transcription factors and chemokine receptors [14, 34]. To investigate, if *Id3*^lo^ Treg cells were enriched for a specific subtype of Treg cells, we analyzed which transcription factors were differentially expressed in *Id3*^lo^ compared to *Id3*^hi^ Treg cells. RNA-sequencing analysis revealed that 10 transcriptional regulators were preferentially expressed in *Id3*^hi^ cells and 30 transcriptional regulators were significantly higher expressed in *Id3*^lo^ Treg cells (Figure 3a). Interestingly, genes enriched in *Id3*^lo^ Treg cells included *Prdm1*, encoding for BLIMP1, which is pivotal for effector Treg cell functions [18], confirming the enrichment of effector Treg cells among the *Id3*^lo^ Treg subpopulation. Indeed, the transcriptional signature of *Id3*^hi^ versus *Id3*^lo^ Treg cells strongly correlated with the transcriptional profile of *Prdm1*^-^ versus *Prdm1*^+^ Treg cells [35] (Figure 3b) suggesting that *Id3*^lo^ and *Prdm1*^+^ Treg cells are highly similar Treg cell populations. The Th1-specific transcription factor *Tbx21* (T-bet) and the Th17-specific transcription factors *Rorc* (RORγ), *Arnt2* (ARNT2) and *Rorα* (ROR*α*) were highly expressed in the *Id3*^lo^ Treg cell population (Figure 3a). Accordingly, genes up-regulated in Th1-specific CXCR3^+^ Treg cells were highly expressed in *Id3*^lo^ Treg cells, whereas genes down-regulated in CXCR3^+^ Treg cells were mainly expressed in *Id3*^hi^ Treg cells (Supplementary Figure 1c) [19]. The Th2- and Tfh-specific transcription factors *Gata3, Irf4* and *Bcl6* were not differentially expressed between *Id3*^lo^ and *Id3*^hi^ Treg cells (data not shown), but IRF4-dependent genes [36] were selectively expressed in *Id3*^lo^ Treg cells (Supplementary Figure 1d). According to their transcription factor profile, *Id3*^lo^ Treg cells had high expression levels of chemokine receptors associated with Treg cell subsets specialized to target Th1 (CCR5, CXCR3, CXCR6), Th17 (CCR4, CCR2, CXCR6) or Th2 (CCR3) immune responses (Figure 3c). *Id3*^lo^ Treg cells expressed high levels of the Treg cell effector molecules *Fgl2*, *Gzmb* (Granzyme B) and *Ebi3* (IL17B) (Figure 3d) and had increased expression of the coinhibitory surface receptors *Icos* and *Lag3* (Figure 3d) providing further support for the effector nature of *Id3*^lo^ Treg cells. However, *Id3*^lo^ Treg cells seemed to express a broad spectrum of effector molecules, targeting several Th cell responses. To further investigate this, we compared the suppressive capacity of CD44^hi^*Id3*^hi^ and CD44^hi^*Id3*^lo^ Treg cells towards Th cell proliferation and differentiation after exclusion of naive-like CD44^lo^*Id3*^hi^ Treg cells. We co-cultured the purified Treg cell populations with CFSE-labeled CD4^+^CD25^-^ effector T (Teff) cells under differentiation conditions for Th1, Th2 and Th17 cells. CD44^hi^*Id3*^lo^ Treg cells more effectively inhibited the proliferation of Teff cells compared to CD44^hi^*Id3*^hi^ Treg cells irrespective of the cytokine milieu (Figure 3e). Furthermore, CD44^hi^*Id3*^lo^ Treg cells were superior in inhibiting the differentiation of Th1, Th2 and Th17 cells as shown by lower percentages of IFNγ^+^, IL13^+^ and IL17A^+^ Teff cells in the presence of *Id3*^lo^ Treg cells (Figure 3f). Taken together, CD44^hi^*Id3*^lo^ Treg cells are highly suppressive Treg cells and not limited to targeting one specific Th cell response.

### Viral infection promotes the differentiation of CD44^hi^*Id3*^hi^ into CD44^hi^*Id3*^lo^ Treg cells

**Figure 4:**
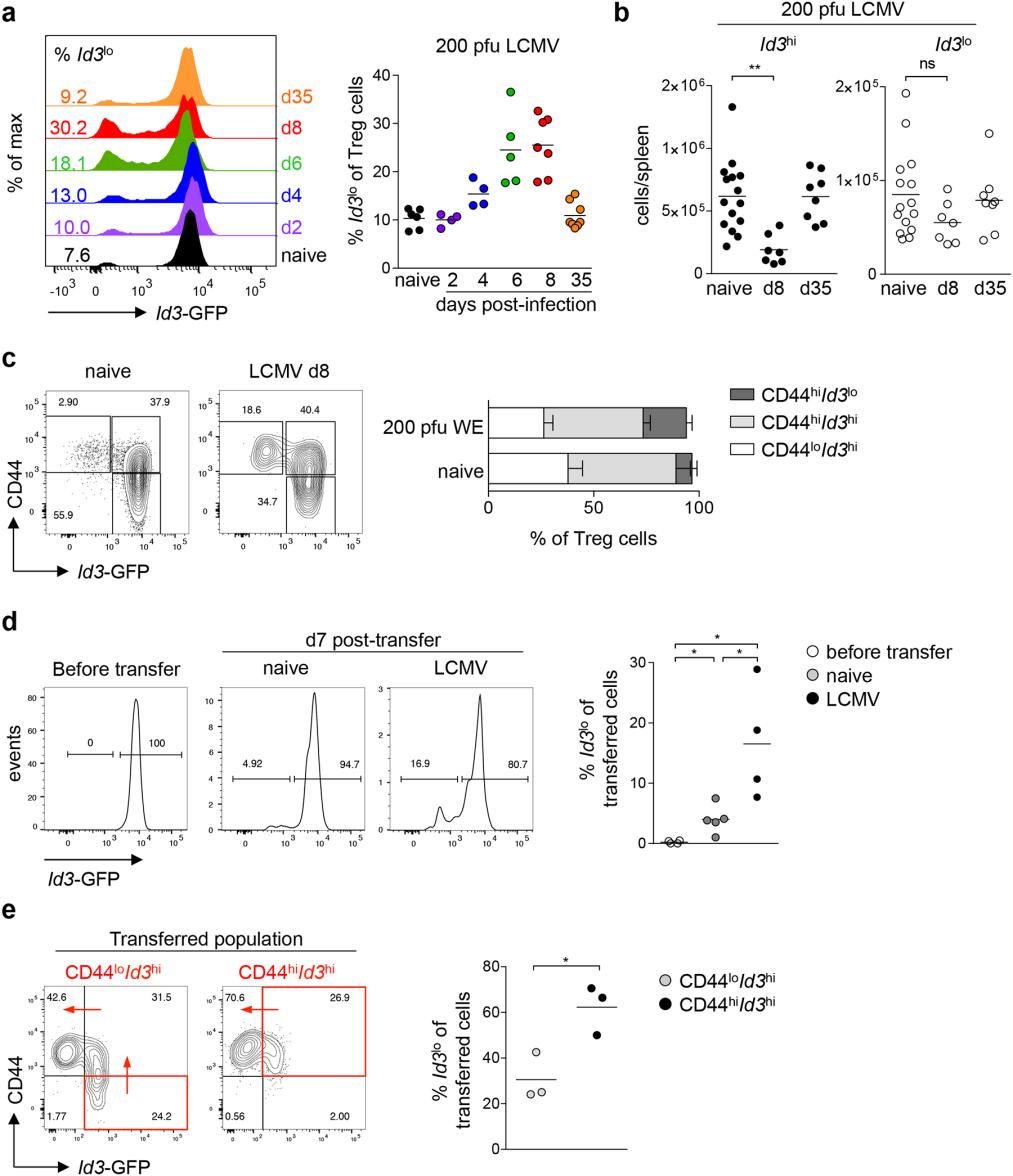
Viral infection promotes the differentiation of CD44^hi^*Id3*^hi^ to CD44^hi^*Id3*^lo^ Treg cells **(a)** *Id3* expression in splenic Treg cells (CD4^+^CD25^+^CD45RB^lo^) from naive *Id3*^GFP/+^ mice and *Id3*^GFP/+^ mice at the indicated times upon 200 pfu LCMV WE infection using flow cytometry (left) and quantification of the percentage of *Id3*^lo^ cells among splenic Treg cells (right). Each symbol represents an individual mouse and horizontal lines are the mean. **(b)** Cell number of *Id3*^hi^ (left) or *Id3*^lo^ (right) Treg cells (CD4^+^CD25^+^CD45RB^lo^) in spleens of naive, d8 or d35 LCMV WE (200 pfu) infected *Id3*^*GFP/+*^ mice. Each symbol represents an individual mouse and horizontal lines are the mean. ^**^p < 0.01; ns = not significant (unpaired Student’s t test). **(c)** *Id3* and CD44 expression in splenic Treg cells (CD4^+^CD25^+^) and quantification of the percentages of CD44^lo^*Id3*^hi^, CD44^hi^*Id3*^hi^ and CD44^hi^*Id3*^lo^ cells among splenic Treg cells of naive or d8 LCMV WE infected mice. Mean ± SEM from at least 3 individual mice are shown. **(d)** *Id3*^hi^ Treg cells (CD4^+^CD25^+^CD45RB^lo^) from naive *Id3*^GFP/+^ mice (Thy1.2) were transferred into wt mice (Thy1.1). One day after, mice were left untreated or infected with 200 pfu LCMV WE. *Id3* expression of purified *Id3*^hi^ Treg cells immediately before transfer and of transferred cells (Thy1.2^+^) 7 days post-transfer in spleens of naive or LCMV WE infected mice (left). Quantification of the percentage of *Id3*^lo^ cells among splenic transferred cells (right). Each symbol represents the mean of 1-3 mice obtained from 4 independent experiments and horizontal lines are the mean. ^*^p < 0.05 (unpaired Student’s t test). **(e)** CD44^lo^*Id3*^hi^ and CD44^hi^*Id3*^hi^ Treg cells (CD4^+^CD25^+^CD45RB^lo^) from naive *Id3*^GFP/+^ mice (Thy1.2) were transferred into wt mice (Thy1.1). One day after, mice were infected with 200 pfu LCMV Docile. *Id3* and CD44 expression of transferred cells (Thy1.2^+^) in spleens were analyzed 7 days post-transfer (left). Quantification of the percentage of *Id3*^lo^ cells among splenic transferred cells (right). For clarity the quadrant of the transferred cell population is marked in red and red arrows indicated the differentiation pathway. Each symbol represents one individual mouse obtained from 2 independent experiments and horizontal lines are the mean.

Inflammation and infection promote the differentiation of Treg cells into specialized effector Treg cells [3]. In this context, IL2 and proinflammatory cytokines increase the abundance of suppressive BLIMP1^+^ Treg cells [18]. Thus, we hypothesized that the proportion of *Id3*^lo^ to *Id3*^hi^ Treg cells should change upon infection. We analyzed *Id3* expression in Treg cells upon LCMV infection, since Treg cells critically affect the antiviral response against LCMV [4, 7, 8]. Infection of *Id3*^GFP/+^ mice with 200 pfu LCMV WE resulted in a transient increase in the relative abundance of *Id3*^lo^ to *Id3*^hi^ Treg cells (Figure 4a). At the peak of the CD8^+^ T cell response, day 8 post-infection, about 25% of Treg cells were *Id3*^lo^ and after virus clearance, on day 35 post-infection, percentages of *Id3*^lo^ and *Id3*^hi^ Treg cells were comparable to naive mice (Figure 4a). In line with previous studies [4], we confirmed that Treg cell numbers transiently declined during acute LCMV infection (Supplementary Figure 2a). Interestingly, this decline in Treg cell numbers was reflected as a selective loss of *Id3*^hi^ Treg cells, whereas *Id3*^lo^ Treg cell numbers were not significantly reduced (Figure 4b). Closer analysis of the relative abundance of CD44^lo^, CD44^hi^*Id3*^hi^ and CD44^hi^*Id3*^lo^ Treg cells showed that the fraction of the quiescent CD44^lo^ Treg cells decreased upon LCMV infection. Strikingly, within the CD44^hi^ Treg cell population, we found a selective increase of CD44^hi^*Id3*^lo^ Treg cells, whereas the relative abundance of CD44^hi^*Id3*^hi^ Treg cells remained unchanged (Figure 4c). These data suggest that viral infection promotes the differentiation of Treg cells into the CD44^hi^*Id3*^lo^ subpopulation.

To determine the lineage relationship between *Id3*^hi^ and *Id3*^lo^ Treg cells, we isolated *Id3*^hi^ and *Id3*^lo^ Treg cells from naive *Id3*^GFP/+^ mice and transferred them into wild-type (wt) recipients. Seven days post-transfer, about 5% of transferred *Id3*^hi^ Treg cells had differentiated into *Id3*^lo^ Treg cells (Figure 4d). Interestingly, LCMV infection one day after Treg cell transfer led to an increased differentiation of transferred *Id3*^hi^ Treg cells into *Id3*^lo^ Treg cells, resulting in about 20% of *Id3*^lo^ Treg cells at day 7 post-transfer (Figure 4d). In contrast, we were not able to detect any *Id3*^hi^ Treg cells in recipients that had received *Id3*^lo^ Treg cells (Supplementary Figure 2b). Next, we separated the *Id3*^hi^ Treg cell population based on differential CD44 expression and found a linear differentiation path from CD44^lo^*Id3*^hi^ over CD44^hi^*Id3*^hi^ to CD44^hi^*Id3*^lo^ Treg cells seven days post-transfer during LCMV infection (Figure 4e). Taken together, CD44^hi^*Id3*^lo^ Treg cells are generated from CD44^hi^*Id3*^hi^ Treg cells and this differentiation is augmented upon viral infection.

### IL2 signaling stimulates the differentiation of *Id3*^hi^ into *Id3*^lo^ Treg cells

**Figure 5:**
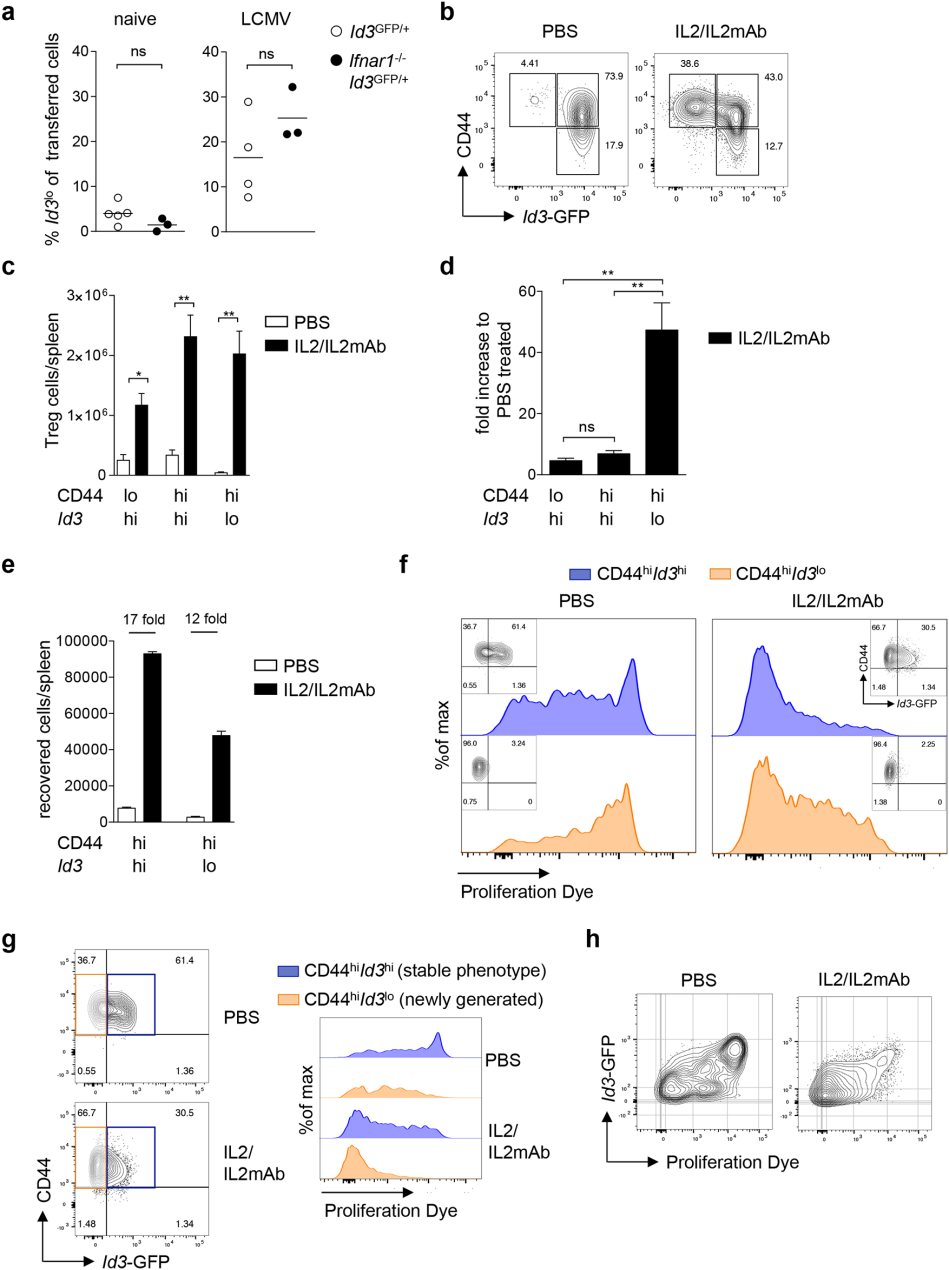
Differentiation into *Id3*^lo^ Treg cells is promoted by IL2 signaling **(a)** *Id3*^hi^ Treg cells (CD4^+^CD25^+^CD45RB^lo^) purified from naive *Id3*^GFP/+^ or *Ifnar1*^-/-^*Id3*^GFP/+^ mice (Thy1.2) were transferred into wt mice (Thy1.1). One day after transfer, mice were left untreated or infected with 200 pfu LCMV WE. The percentage of *Id3*^lo^ Treg cells among transferred cells (Thy1.2^+^) 7 days post-transfer in spleens of naive or LCMV WE infected host mice. Results from 1-3 mice per experiment were pooled and the mean value from each of 3-5 independent experiments is presented by the symbols. The horizontal lines are the mean of all experiments. ns = not significant (unpaired Student’s t test). **(b)** *Id3* and CD44 expression in splenic Treg cells (CD4^+^CD25^+^) of PBS or IL2/IL2mAb treated *Id3*^GFP/+^ mice at day 5 after treatment. **(c)** Total cell number of CD44^lo^*Id3*^hi^, CD44^hi^*Id3*^hi^ and CD44^hi^*Id3*^lo^ Treg cells (CD4^+^CD25^+^) in spleens of PBS or IL2/IL2mAB treated mice. Mean ± SEM from at least 4 independent experiments are shown. ^*^p < 0.05, ^**^p < 0.01 (unpaired Student’s t test). **(d)** Fold increase in CD44^lo^*Id3*^hi^, CD44^hi^*Id3*^hi^ and CD44^hi^*Id3*^lo^ Treg cells in IL2/IL2mAb treated mice compared to PBS treated mice. Mean ± SEM from at least 4 independent experiments are shown. ^**^p < 0.01; ns = not significant (unpaired Student’s t test). **(e-h)** CD44^hi^*Id3*^hi^ and CD44^hi^*Id3*^lo^ Treg cells (CD4^+^CD25^+^) from IL2/IL2mAb treated *Id3*^GFP/+^ mice (Thy1.2) were labeled with a proliferation dye and transferred into wt mice (Thy1.1). Transferred cells (Thy1.2^+^) were analyzed 7 days post-transfer in the spleens after PBS or IL2/IL2mAB treatment. (e) Absolute numbers of recovered Thy1.2^+^ cells. (f) Representative analysis of *Id3* and CD44 expression (small insets) and proliferation of Thy1.2^+^ cells recovered after transferring CD44^hi^*Id3*^hi^ (blue) or CD44^hi^*Id3*^lo^ (orange) Treg cell populations. (g) *Id3* and CD44 expression of recovered Thy1.2^+^ cells in spleens of PBS or IL2/IL2mAB treated mice that had received CD44^hi^*Id3*^hi^ Treg cells (left). Representative proliferation analysis of recovered phenotypically stable Thy1.2^+^CD44^hi^*Id3*^hi^ (blue) or newly generated Thy1.2^+^CD44^hi^*Id3*^lo^ (orange) cells in spleens of PBS or IL2/IL2mAB treated mice that had received CD44^hi^*Id3*^hi^ Treg cells (right). (h) Representative analysis of *Id3* expression versus proliferation of recovered Thy1.2^+^ cells in spleens of PBS or IL2/IL2mAB treated mice that had received CD44^hi^*Id3*^hi^ Treg cells.

Type I interferons (IFN), abundantly present in the early stages of LCMV infection, inhibit Treg cell proliferation and promote Treg cell activation [4, 37, 38]. Thus, we hypothesized that type I IFNs could play a role in the differentiation of *Id3*^hi^ to *Id3*^lo^ Treg cells. *Id3*^hi^ Treg cells isolated from control *Id3*^GFP/+^ or from *Ifnar1*^-/-^*Id3*^GFP/+^ mice, lacking the type I IFN receptor, were transferred into wt recipients and *Id3* expression was analyzed seven days post-transfer. Transferred *Ifnar1*^-/-^*Id3*^hi^ Treg cells differentiated into *Id3*^lo^ Treg cells to the same extent as transferred control *Id3*^hi^ Treg cells in naive recipient mice (Figure 5a, left). Again, in LCMV infected host mice a greater percentage of *Id3*^hi^ Treg cells differentiated into *Id3*^lo^ Treg cells than in naive mice, however this was independent of type I IFN signaling (Figure 5a, right).

It has been shown that interleukin-2 (IL2) signaling induces the differentiation into BLIMP1^+^ effector Treg cells [18]. To test whether the differentiation of *Id3*^hi^ to *Id3*^lo^ Treg cells was triggered by IL2, we induced IL2 signaling by injecting complexes of IL2 and anti-IL2 antibody (IL2/IL2mAb) [39] into *Id3*^GFP/+^ reporter mice and analyzed splenic Treg cells. We found a strong increase in the relative abundance of *Id3*^lo^ Treg cells upon IL2/IL2mAb treatment (Figure 5b). Absolute numbers of all Treg cell subsets increased upon treatment (Figure 5c), yet the fold increase in the CD44^hi^*Id3*^lo^ Treg cell population was 7 to 10-fold higher than in the CD44^lo^*Id3*^hi^ or CD44^hi^*Id3*^hi^ Treg cell populations (Figure 5d). This suggests that, beside its function in stimulating Treg cell proliferation, IL2 signaling induces the differentiation of *Id3*^hi^ into *Id3*^lo^ Treg cells.

To further delineate the role of IL2 on proliferation and differentiation of *Id3*^lo^ and *Id3*^hi^ Treg cells, CD44^hi^*Id3*^lo^ or CD44^hi^*Id3*^hi^ Treg cells were labeled with a proliferation dye and equal numbers were transferred into wt recipients, which were injected with IL2/IL2mAb complexes or PBS as control. Cell recovery was lower in mice that received CD44^hi^*Id3*^lo^ compared to CD44^hi^*Id3*^hi^ Treg cells (Figure 5e, white bars). Cell numbers increased upon IL2/IL2mAb treatment in both cases, but to a greater extend in mice that received CD44^hi^*Id3*^hi^ Treg cells indicating a higher proliferative capacity of this Treg cell population (Figure 5e). Transferred CD44^hi^*Id3*^lo^ Treg cells remained CD44^hi^*Id3*^lo^ also upon IL2/IL2mAb treatment, while transferred CD44^hi^*Id3*^hi^ Treg cells differentiated to a great extend into CD44^hi^*Id3*^lo^ cells (Figure 5f, small insets). CD44^hi^*Id3*^hi^ Treg cells and their CD44^hi^*Id3*^lo^ progeny proliferated moderately more than phenotypically stable CD44^hi^*Id3*^lo^ Treg cells (Figure 5f), explaining the observed increase in cell recovery. IL2/IL2mAb treatment greatly induced proliferation of CD44^hi^*Id3*^hi^ and CD44^hi^*Id3*^lo^ Treg cells (Figure 5f). Comparison of newly generated CD44^hi^*Id3*^lo^ Treg cells and those that remained CD44^hi^*Id3*^hi^ at day 7 post-transfer (stable phenotype) showed that the newly differentiated CD44^hi^*Id3*^lo^ Treg cells had undergone more cell divisions (Figure 5g). Reduced *Id3* expression correlated with increased numbers of cell divisions and this effect was enhanced by IL2/IL2mAb treatment (Figure 5h). Taken together, the differentiation of *Id3*^hi^ into *Id3*^lo^ Treg cells was accompanied by proliferation and induced by IL2.

### Highly suppressive expression profile of *Id3*^lo^ Treg cells during infection

**Figure 6:**
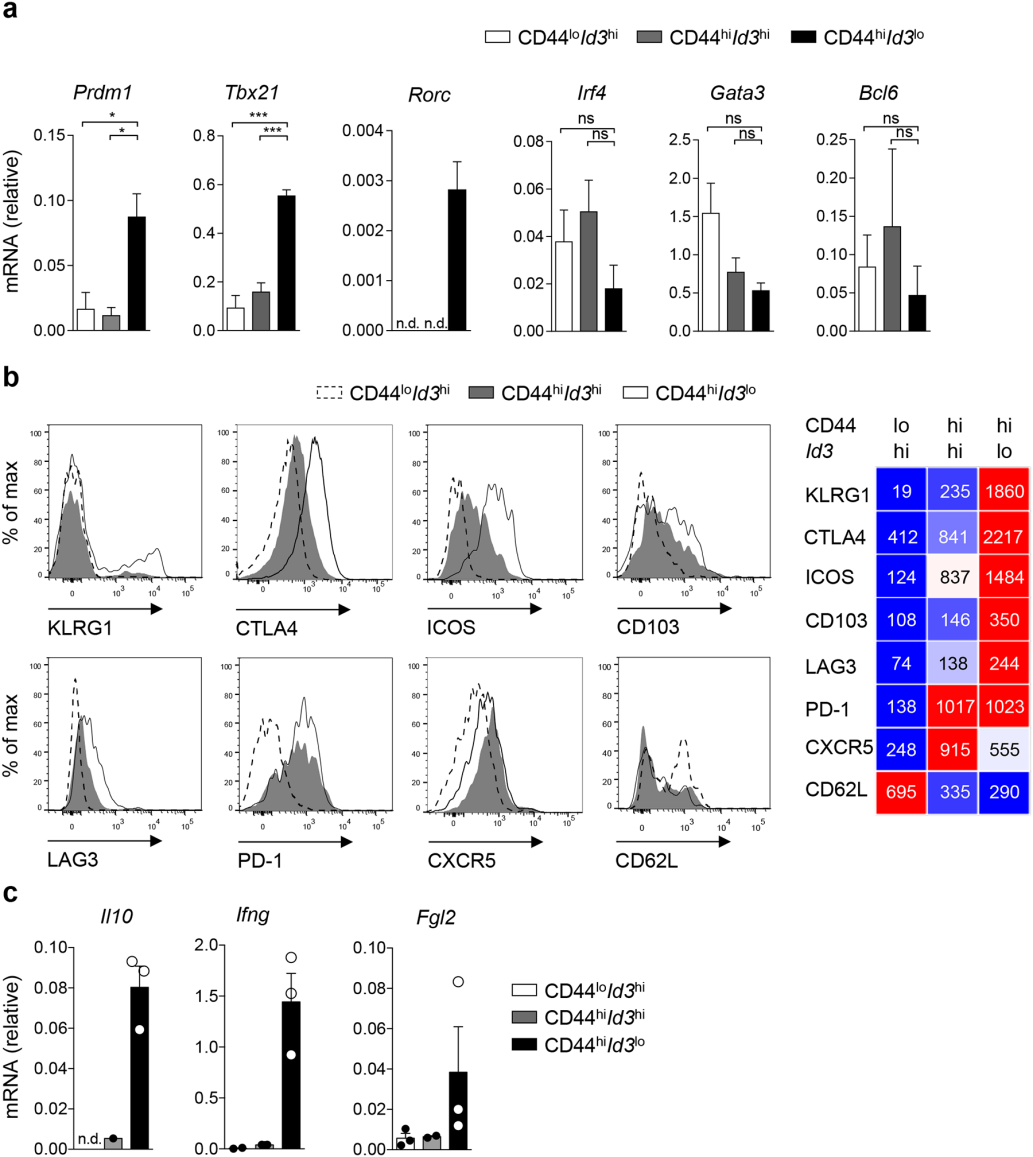
*Id3*^lo^ Treg cells are highly suppressive effector Treg cells after infection **(a, c)** Quantitative RT-PCR analysis of the indicated mRNA transcripts in CD44^lo^*Id3*^hi^ (white), CD44^hi^*Id3*^hi^ (grey) or CD44^hi^*Id3*^lo^ (black) splenic Treg cells (CD4^+^CD25^+^) from d8 LCMV WE infected *Id3*^GFP/+^ mice. (a) Mean ± SEM from 3 individual mice or (c) mean ± SEM and single values from 3 individual mice are shown. n.d. = not detectable in all experiments, ^*^p < 0.05; ^**^p < 0.01; ns = not significant (unpaired Student’s t test). **(b)** Flow cytometric analysis of the indicated surface markers on CD44^lo^*Id3*^hi^, CD44^hi^*Id3*^hi^, CD44^hi^*Id3*^lo^ Treg cells (CD4^+^CD25^hi^) of d8 LCMV WE infected *Id3*^GFP/+^ mice or in the case of CTLA4 of IL2/IL2mAB treated *Id3*^GFP/+^ mice. Representative histograms (left) and quantification of the mean fluorescence intensity of the indicated surface proteins from 3 individual mice (right) are shown.

Next, we analyzed the transcriptional profile of *Id3*^lo^ Treg cells under inflammatory conditions. As controls, we additionally separated CD44^lo^I*d3*^hi^ from CD44^hi^*Id3*^hi^ Treg cells to prevent skewing of the analysis. Isolated CD44^lo^*Id3*^hi^, CD44^hi^*Id3*^hi^ and CD44^hi^*Id3*^lo^ Treg cells from LCMV infected *Id3*^GFP/+^ mice on day 8 post-infection were analyzed for the expression of transcription factors and suppressive molecules by quantitative RT-PCR and FACS. Significantly higher levels of *Prdm1* were detected in CD44^hi^*Id3*^lo^ Treg cells compared to CD44^hi^*Id3*^hi^ Treg cells, suggesting that also BLIMP1 expression separates CD44^hi^ Treg cells into two distinct populations (Figure 6a and Supplementary Figure 3a). Furthermore, we found increased mRNA levels of *Tbx21* and *Rorc* in *Id3*^lo^ Treg cells, but no significant differences in the expression of the Th2-specific transcription factors *Gata3* and *Irf4* or the Tfh-specific transcription factor *Bcl6* were found between the analyzed populations (Figure 6a and Supplementary Figure 3a). These data confirmed that certain specialized Treg cells, such as those targeting Th1 and Th17 cells are enriched among *Id3*^lo^ Treg cells, while follicular Treg cells are mostly found within the *Id3*^hi^ Treg cell population. This notion was enforced by higher CXCR5^+^ expression in the CD44^hi^*Id3*^hi^ Treg cell population (Figure 6b and Supplementary Figure 3b). The CD44^hi^*Id3*^lo^ Treg cell population could be further divided by low/absent and high surface expression of KLRG1 (Figure 6b). CD44^hi^*Id3*^lo^ Treg cells had selectively higher expression of CTLA4 and ICOS, slightly higher CD103 and LAG3 expression and similar PD1 and CD62L surface levels compared to the CD44^hi^*Id3*^hi^ Treg cell population. With regard to their suppressive function, RT-PCR analysis showed that expression of the cytokines *Il10* and *Ifng*, as well as the immunosuppressive molecule *Fgl2* was mostly restricted to the CD44^hi^*Id3*^lo^ Treg cell population (Figure 6c and Supplementary Figure 3a). Altogether, these data suggest that during infection the CD44^hi^*Id3*^lo^ Treg cell population is the main producer of suppressive molecules.

### *Id3*^lo^ Treg cells are more abundant during chronic compared to acute viral LCMV infection

**Figure 7:**
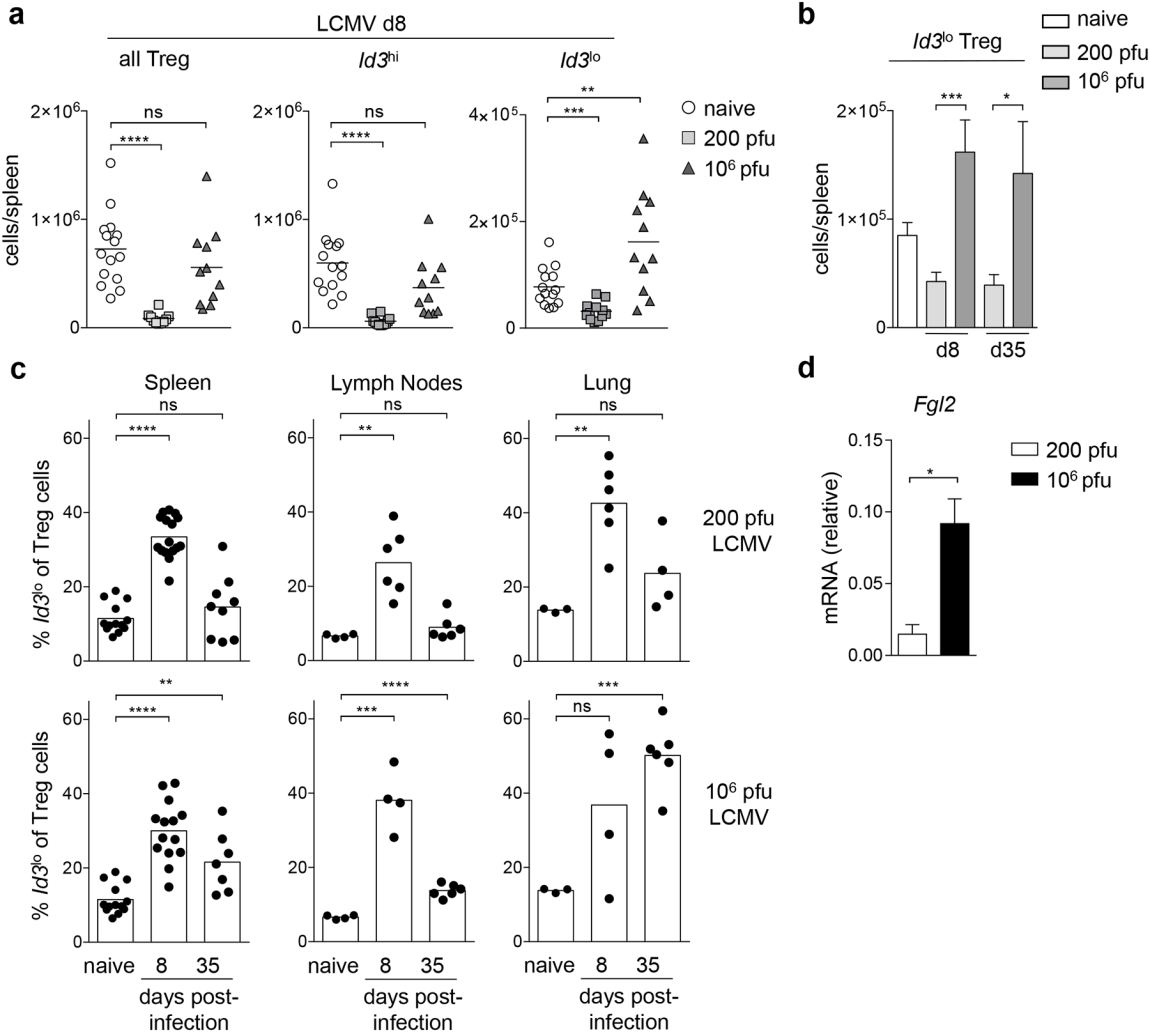
*Id3*^lo^ Treg cells are more abundant in chronic LCMV infection **(a)** Cell number of all, *Id3*^hi^ or *Id3*^lo^ Treg cells (CD4^+^CD25^+^CD45RB^lo^) in spleens of naive, 200 (acute) or 10^6^ (chronic) pfu LCMV Docile infected *Id3*^GFP/+^ mice. Each symbol represents an individual mouse and horizontal lines are the mean. ^**^p < 0.01; ^***^p < 0.001; ^****^p < 0.0001; ns = not significant (unpaired Student’s t test). **(b)** *Id3*^lo^ Treg cell numbers per spleen upon infection with 200 (acute) or 10^6^ (chronic) pfu LCMV Docile at d8 and d35 post-infection. Mean ± SEM from at least 4 individual mice are shown. ^*^p < 0.05; ^***^p < 0.001 (unpaired Student’s t test). **(c)** *Id3*^lo^ Treg cell percentages among Treg cells (CD4^+^CD25^+^) from the spleens, lymph nodes and lungs of naive or infected mice with 200 (acute, top) or 10^6^ pfu (chronic, bottom) LCMV Docile at d8 and d35 post-infection. Each symbol represents an individual mouse and bars are the mean. ^**^p < 0.01; ^***^p < 0.001; ^****^p < 0.0001; ns = not significant (unpaired Student’s t test). **(d)** Quantitative RT-PCR analysis of *Fgl2* mRNA transcripts in CD44^hi^*Id3*^lo^ Treg cells (CD4^+^CD25^+^) from 200 (white) or 10^6^ (black) pfu LCMV Docile infected *Id3*^GFP/+^ mice at d8 post-infection. Mean ± SEM from 3 independent experiments are shown. ^*^p < 0.05 (unpaired Student’s t test).

We next investigated whether the abundance of the highly suppressive *Id3*^lo^ Treg cell subset might be differentially elevated in acute compared to chronic infection. We infected *Id3*^GFP/+^ mice with either 200 pfu LCMV Docile for an acute infection or 10^6^ pfu LCMV Docile for a chronic infection [27] and analyzed the abundance of Treg cell populations. At day 8 post-infection with 200 pfu LCMV Docile, overall Treg cells, and specifically both *Id3*^lo^ and *Id3*^hi^ Treg cell numbers decreased (Figure 7a). In contrast, at day 8 post-infection with 10^6^ pfu LCMV Docile, overall Treg cell numbers were comparable to naive mice while *Id3*^lo^ Treg cell numbers increased (Figure 7a). The absolute abundance of *Id3*^lo^ Treg cells drastically changed, resulting in a 2-fold increase of *Id3*^lo^ Treg cells compared to naive mice and about 4-fold compared to acutely infected mice at day 8 and day 35 post-infection (Figure 7b). This increased abundance of *Id3*^lo^ Treg cells among the Treg cell pool was observed in the spleen, lymph nodes and lung at day 8 both under acute or chronic infection (Figure 7c). Strikingly, only during chronic infection the increased abundance of *Id3*^lo^ Treg cells persisted until day 35 upon infection in the spleen, and most prominently in the lungs (Figure 7c, lower panel). The overall phenotype of *Id3*^lo^ Treg cells was comparable in acute and chronic infected mice (Supplementary Figure 3a, 3b). However, it was striking that during chronic infection *Id3*^lo^ Treg cells produced significantly higher levels of the immunosuppressive cytokine *Fgl2* compared to *Id3*^lo^ Treg cells in acute infected mice (Figure 7d). In summary, *Id3*^lo^ Treg cells expressing elevated levels of *Fgl2* are highly abundant in mice with chronic LCMV infection.

## DISCUSSION

In this study, we identified a highly suppressive Treg cell subpopulation characterized by low expression of the transcriptional regulator *Id3*, which (1) defined a subpopulation of activated CD44^hi^ Treg cells, (2) had a transcriptional profile of late-stage differentiated effector Treg cells, (3) expressed high levels of inhibitory surface receptors and immunosuppressive cytokines and (4) was superior in suppressing proliferation and differentiation of T cells compared to CD44^hi^*Id3*^hi^ Treg cells. *Id3*^lo^ Treg cells were the predominant Treg cell population during chronic LCMV infection. Therefore, we suggest that *Id3*^lo^ Treg cells contribute to the dysregulated T cell response during chronic infection and propose that preventing the differentiation of *Id3*^hi^ into *Id3*^lo^ Treg cells might provide a new therapeutic approach for treating chronic infections.

To fulfill diverse functions, from controlling self-reactive T cells to preventing or allowing pathogen and tissue-specific immune responses, different subpopulations of Treg cells utilize different mechanisms to regulate the immune response [14, 15]. CD44^hi^ Treg cells, which are generated by TCR signaling from naive-like Treg cells, possess greater suppressive activity against effector T cells compared to the naive-like Treg cell population [33, 40]. We show here, that this activated CD44^hi^ Treg cell population can be further divided into *Id3*^lo^ and *Id3*^hi^ populations. While the CD44^hi^*Id3*^hi^ population already had some features of effector Treg cells, such as increased surface levels of CD103 and intermediate ICOS levels, the levels of mRNA transcripts of the immunomodulatory cytokines *Il10* and *Fgl2* and of the lineage determining transcription factors *Prdm1*, *Tbx21* and *Rorc* were similar to naive-like CD44^lo^ Treg cells. These data suggest that CD44^hi^*Id3*^hi^ Treg cells, although based on elevated expression of the surface marker CD44 already categorized as activated Treg cells, have only a limited set of suppressor functions and retain the capacity to further specialize.

Further specialization tailors Treg cells to specifically suppress Th1, Th2, Th17 or Tfh cell responses [14, 34]. CD44^hi^*Id3*^lo^ Treg cells were highly suppressive towards any T cell response tested, suggesting that this population is enriched with cells possessing great suppressive capacity towards a variety of T cell responses. Highly suppressive effector Treg cells have been described to express high levels of the transcription factor BLIMP1 [18]. Accordingly, also CD44^hi^*Id3*^lo^ Treg cells had increased *Prdm1* expression and based on their gene expression profiles *Id3*^lo^ and BLIMP1^+^ Treg cells were highly similar Treg cell subpopulations. However, it was suggested that BLIMP1^+^ effector Treg cells also include follicular Treg cells [14, 41] and our data suggests that follicular Treg cells were not specifically included in the CD44^hi^*Id3*^lo^ Treg cell population. Thus, we propose that *Id3*^lo^ Treg cells are a subpopulation of BLIMP1^+^ effector Treg cells, including a wide range, but not all types of specialized effector Treg cells. Mechanistically, we propose that BLIMP1 is up-regulated in Treg cells differentiating into effector Treg cells and subsequently, in a subset of effector Treg cells, BLIMP1 acts to suppress *Id3* expression, as it has been shown to directly repress *Id3* transcription in CD8^+^ T cells [42].

Treg cell differentiation in infection is controlled by different signals, such as TCR and cytokine signaling, with TCR signaling being required for the differentiation of naive-like to activated CD44^hi^ Treg cells [12, 33, 40]. We propose here that the differentiation into highly suppressive *Id3*^lo^ Treg cells occurs in a multi-step process. First, antigen-mediated TCR signaling activates naive-like Treg cells, resulting in the generation of activated *Id3*^hi^CD44^hi^ Treg cells with a limited suppressive potential. In a later step, *Id3* is down-regulated, possibly through BLIMP-1 mediated repression, resulting in further differentiation and specialization of Treg cells. This later step is augmented upon LCMV infection, and it is most probably triggered by extracellular signals, among others IL2. IL2 has multiple effects on Treg cells, ranging from inducing Treg cell specific gene expression, such as *Foxp3,* to supporting Treg cell homeostasis and proliferation [43–45]. IL2 is also required for the development of highly suppressive KLRG1^+^ Treg cells [46] and of BLIMP1^*+*^ effector Treg cells [18]. Our data demonstrated that IL2 signaling promoted the accumulation of *Id3*^lo^ Treg cells. This preferential accumulation may have resulted exclusively from IL2 promoted differentiation of *Id3*^hi^ into *Id3*^lo^ Treg cells or additionally from IL2-mediated selective expansion or/and survival of the CD44^hi^*Id3*^lo^ Treg cell subpopulation. IL2/IL2mAb complex treatment, for instance, stimulates KLRG1^-^ Treg cells to proliferate and differentiate into KLRG1^+^ Treg cells, whereas proliferation of KLRG1^+^ Treg cells is not induced [46]. In a similar way, IL2/IL2mAb complex treatment stimulated *Id3*^hi^ Treg cells to proliferate and develop into *Id3*^lo^ Treg cells, but it also stimulated proliferation of purified *Id3*^lo^ Treg cells. In combination with our data showing that the fold increase of *Id3*^lo^ Treg cells was much greater than that of the *Id3*^hi^ Treg cell subsets upon IL2/IL2mAb complex treatment, we suggest that besides supporting proliferation of both *Id3*^lo^ and *Id3*^hi^ Treg cell subsets, IL2 also drives differentiation into *Id3*^lo^ Treg cells. Regarding its role as a survival factor, our RNA-sequencing data showed that *Id3*^lo^ Treg cells expressed low levels of the anti-apoptotic protein Bcl-2, suggesting that these cells are probably short-lived compared to *Id3*^hi^ Treg cells. Indeed, cell recovery after transfer of *Id3*^lo^ Treg cells was reduced compared to *Id3*^hi^ Treg cells. As this trend was unaltered after IL2/IL2mAb complex treatment, a role of IL2 specifically supporting survival of *Id3*^lo^ Treg cells is unlikely. Regardless of its exact way of action, we clearly show that high-dose IL2 treatment led to increased abundance of highly suppressive *Id3*^lo^ Treg cells. Thus, our data suggest that IL2 treatment differentially affects the abundance of specialized Treg cell subtypes. Thus, it would be interesting to determine, if there is a relationship between applied IL2/IL2mAb complex dose and predominant Treg cell phenotype that can be monitored in clinical applications of IL2, especially since the effects of low versus high dose IL2 treatment are currently investigated for the treatment of autoimmune diseases such as diabetes [47, 48].

We showed here for the first time that a subset of effector Treg cells in a late stage of differentiation was characterized by low *Id3* expression. Other T cell differentiation processes are also accompanied by the down-regulation of *Id3*. For instance, high Id3 expression is required for the maintenance of the naive phenotype of T cells [24] and *Id3*^lo^CD8^+^ T cells differentiate into KLRG1^+^ effector cells in infection [26, 27]. In Treg cells, low *Id3* expression could have several functional consequences: (1) Id3 acts by inhibiting the gene regulatory activity of the E protein transcription factors E2A and HEB [21], and known E2A target genes in T cells include *Il10* and *Rorct* [49, 50], suggesting that the down-regulation of *Id3* in activated Treg cells is necessary to activate the expression of effector molecules and lineage determining factors by releasing E protein activity; (2) Id3 maintains the expression of Foxp3 in Treg cells [29], however, Foxp3 transcript and protein expression were not reduced in *Id3*^lo^ compared to *Id3*^hi^ Treg cells. We suggest that in activated Treg cells feedback mechanisms prevent the down-regulation of Foxp3 expression by increased E47 activity. One of such mechanisms could be the increased *Id2* expression we found by RNA-Sequencing in *Id3*^lo^ Treg cells (Figure 3a), which would guarantee low E protein activity. And (3) loss of both Id3 and Id2 expression in Treg cells results in the preferential generation of CXCR5^+^ follicular Treg cells [28], whereas we found that follicular Treg cells were mainly included in the CD44^hi^*Id3*^hi^ Treg cell population. Thus, we propose that again high levels of Id2 in the *Id3*^lo^ Treg cell population prevent the differentiation into follicular Treg cells. Further, since follicular Treg cells were generated within the CD44^hi^*Id3*^hi^ Treg cell population, it is tempting to speculate that high Id3 levels do not restrict the generation of follicular Treg cells and thus it would be of uttermost interest to understand which transcriptional targets are selectively controlled by Id2 and Id3 in Treg cells.

Finally, we found an increased abundance of *Id3*^lo^ Treg cells during chronic compared to acute LCMV infection suggesting that the *Id3*^lo^ effector Treg cell population specifically contributed to the insufficient T cell responses during chronic viral infections. During chronic LCMV infection, blocking IL10 receptor signaling or FGL2 depletion results in enhanced antiviral CD8^+^ T cell responses and viral clearance [51, 52]. Since *Id3*^lo^ Treg cells have preferential high expression of *Il10* and *Fgl2*, we propose that the *Id3*^lo^ Treg subpopulation is a major source of IL10 and FGL2 during chronic LCMV infection. LCMV-specific Treg cell numbers decline during acute and chronic infection [53], however, *Id3*^lo^ Treg cells specifically accumulated during chronic infection. We therefore speculate, that *Id3*^lo^ Treg cells were most probably not LCMV-specific, but generated from LCMV-unrelated, maybe even self-reactive, activated CD44^hi^*Id3*^hi^ Treg cells by the action of IL2 and other unknown factors present during infection. These highly suppressive *Id3*^lo^ Treg cells would then suppress the immune response in a bystander mechanism contributing to chronic infection [54]. Most interestingly upon chronic LCMV infection, *Id3*^lo^ Treg cells specifically persisted within the lungs, an organ wherein LCMV-Docile persists for a long time. Thus, the preferential presence of *Id3*^lo^ Treg cells within this organ might be either causative for virus persistence within this site or the consequence of increased virus presence, which would constantly trigger differentiation of *Id3*^hi^ into *Id3*^lo^ Treg cells. Regardless, the increased abundance of highly suppressive *Id3*^lo^ Treg cells would contribute to reduced T cell responses at these sites.

In summary, the present study shows that activated CD44^hi^ Treg cells further differentiate into highly suppressive effector Treg cells. During this differentiation process, Treg cells down-regulate *Id3* and subsequently acquire the expression of transcription factors specific for highly suppressive effector Treg cells. *Id3*^lo^ Treg cells were, indeed, highly suppressive, most likely due to their high ICOS, *Il10* and *Fgl2* expression levels. Interestingly, these highly suppressive *Id3*^lo^ Treg cells were more abundant during chronic compared to acute LCMV infection. Thus, preventing the differentiation of CD44^hi^*Id3*^hi^ to CD44^hi^*Id3*^lo^ or specifically depleting the *Id3*^lo^ Treg cell subset in chronic viral infections might rescue antiviral T cell responses, resulting in viral clearance.

## MATERIALS AND METHODS

### Mice and LCMV infections

*Id3*^GFP/+^ [B6.Id3tm2.1Cmu] [24], Thy1.1 and *Ifnar*^- /-^*Id3*^GFP/+^ [55] mice were housed and bred in specific pathogen-free conditions. All experiments were performed on 7–15-week-old mice. For LCMV experiments, mice were infected i.v. with 200 pfu LCMW-WE or LCMV Docile using 200 pfu for acute and 10^6^ pfu for chronic infection. LCMV was grown in Madin Darby canine kidney (MDCK) cells and titered on MC57G fibrosarcoma cells by standard focus-forming assay as described [56]. Sample sizes were determined based on experience [27]. The groups were neither randomized, nor were experiments conducted in a blinded manner. All animal experiments were approved and performed in accordance with the guidelines of the local animal care and use committees and the Regierungspräsidium Freiburg.

### *In vivo* transfer experiments and IL2/IL2mAb treatment

*Id3*^hi^ or *Id3*^lo^ Treg cells (CD4^+^CD8^-^CD25^+^Thy1.2^+^) were FACS sorted from spleen and LN of *Id3*^*GFP*/+^ or *Ifnar*^- /-^*Id3*^*GFP*/+^ mice. 2.5 x 10^5^ *Id3*^hi^, CD44^hi^*Id3*^hi^, CD44^lo^*Id3*^hi^ or 10^5^ *Id3*^lo^ Treg cells were transferred into wt (Thy1.1^+^) recipients. Recipient mice were infected with 200 pfu LCMV WE or 10^6^ pfu LCMV Docile one day before or after transfer, as indicated. Spleens were analyzed seven days post-transfer. Mice with no detectable Thy1.1^+^ cells were excluded from the analysis. For IL2/IL2mAb treatments 2 μg IL2 (Immunotools) were incubated with 10 μg anti-IL2 antibody (JES6-1A12, ebioscience) in PBS for 30 min at 37°C prior to intraperitoneal injections. *Id3*^*GFP*/+^ mice were treated with IL2/IL2mAb on three consecutive days and analyzed five days after the first treatment.

### *In vitro* suppression assay

10^5^ MACS-isolated or FACS-sorted (CD4^+^CD8^-^CD25^-^CD44^lo^CD62L^hi^; for Th2 differentiation) and CFSE-labeled CD4^+^CD25^-^ cells were cultured with 1 x 10^5^, 0.5 x 10^5^ or 0.25 x 10^5^ FACS sorted *Id3*^hi^ or I*d3*^lo^ Treg cells (CD4^+^CD8^-^CD25^+^CD44^hi^) from spleens of IL2/IL2mAb treated *Id3*^*GFP*/+^ mice in the presence of 2.5 μl (Th1, Th2) or 5 μl (Th17, Th2 differentiation) CD3/CD28 activator beads (life technologies) under the following conditions: Th1: IL-12 (5 ng/ml, Peprotech), anti-IL-4 (10 ng/ml, ebioscience); Th2 (proliferation): IL-4 (20 ng/ml, Peprotech), anti-IL-12 (10 ng/ml, ebioscience), anti-IFN-γ (10 ng/ml, ebioscience); Th2 (differentiation): IL-4 (100 ng/ml, Peprotech), IL2 (50U), anti-IL12 (10 ng/ml, ebioscience), anti-IFNγ (10 ng/ml, ebioscience), anti-CD28 (1μg/ml); Th17: IL-6 (20 ng/ml, Peprotech), TGF-β (5 ng/ml, R&D), anti-IL-4 (10 ng/ml, ebioscience), anti-IFN-γ (10 ng/ml, Peprotech). After 6 days cells were stimulated with PMA and Ionomycin for 1 hour and additionally with Golgi Plug for another 3 hours. The percentage of divided cells was calculated similar to Gett et al. [57]. In detail, relative cell numbers per division were determined using FlowJo and precursor frequencies were calculated as cell number/(2^number of cell divisions). Finally, the percentage of divided cells was calculated as 100^*^((precursor number of cells in divisions 1-6)/(total number of precursor cells)).

### Flow cytometry and cell sorting

Single-cell suspensions for FACS analysis were obtained from the indicated organs. Splenic erythrocytes were eliminated using red blood cell lysis buffer. Cells were stained at 4°C for 15 min in PBS supplemented with 1% fetal calf serum and 2 μM EDTA, and various fluorochrome-conjugated antibodies purchased from eBioscience. Propidium iodide (1 μg/ml; Sigma) was included to exclude dead cells. A three-step CXCR5 staining was performed as described [58] by using purified rat anti-mouse CXCR5 (BD), a secondary Biotin-SP-conjugated Affinipure F(Ab’)2 Goat anti-rat IgG (Jackson Immunoresearch) and finally with streptavidin-eFluor^®^450 (ebioscience). For labeling with CFSE or Proliferation Dye, cells were resuspended at a concentration of 10 × 10^6^ cells per ml in PBS containing 1 μM CFSE (Sigma) or 5 μM Cell Proliferation Dye eFluor^®^670 (ebioscience) and incubated for 10 min at 37°C. For intracellular staining of Foxp3 and CTLA4, cells were fixed and permeabilized using the Foxp3 Staining Buffer Kit (eBioscience), according to the manufacturer’s protocol, after staining of surface antigens and staining with the Fixable Viability Dye (eBioscience; eFluor^®^450 or eFluor^®^780) to exclude dead cells. Data were collected using the LSR Fortessa (BD Bioscience) flow cytometer and were analyzed with FlowJo software (Tree Star). Specific cell populations were either sorted on a MoFlo Astrios (Beckman Coulter), FACSAria III (BD Biosciences) or isolated by magnetic cell sorting (MACS; Miltenyi Biotec) with anti-biotin microbeads according to the manufacturer’s protocol. Antibodies with the following specificities were used: CD4 (RM4-5), TCRβ (H57-597), CD25 (PC61.5), CD45RB (C363.16A), Thy1.1 (HIS51), Thy1.2 (53-2.1), CD62L (MEL-14), Foxp3 (FJK-16s), CD3ε (145-2C11), CD8α (53-6.7), B220 (RA3-6B2), MHCII (M5/114.15.2), CD44 (IM7), KLRG1 (2F1), PD1 (RMP1-30), LAG3 (eBioC9B7W), ICOS (15F9), CXCR5 (2G8), CTLA4 (UC10-4B9), CD103 (2E7), IL17A (17B7), IFNγ (XMG1.2), IL4 (11B11) and NK1.1 (PK136).

### RNA sequencing

*Id3*^hi^ and *Id3*^lo^ Treg cells (CD3^+^CD4^+^CD8^-^CD25^+^CD45RB^lo^) were sorted by flow cytometry from spleens of individual naive *Id3*^*GFP*/+^ mice directly into RLT buffer (Qiagen). Total RNA was isolated using RNAeasy MicroKit (Qiagen) according to the manufacturer’s recommendations. cDNA was generated using Ovation RNA-seq system V2 (NuGEN) and libraries were constructed with Ovation Ultralow System V2 1-16 (NuGEN) according to the manufacturer’s instructions. The libraries were subjected to 50 cycles of NextSeq 500 sequencing from 2-3 samples per cell type. Data analysis was performed with RNAExpress (Illumina), which performed alignment of reads to the mouse reference genome using the STAR aligner (mm10) and determined differentially expressed genes using DESeq2. Genes of interest (fold-change > 4) for the heat maps were handpicked and heat maps were generated with GENE-E (Broad Institute). Analysis of signature genes within the *Id3*^lo^/*Id3*^hi^ comparison used previously determined gene sets: canonical Treg signature [30], T cell activation/proliferation signature from *in vivo* activated T cells [30], genes up-regulated in activated Treg cells [31], IRF4-dependent signature in Treg cells [36], CXCR3^+^ Treg signature [19]. Broad Institute GSEA was performed to determine Normalized Enrichment Scores (NES) and False Discovery Rates (FDR). Gene Set Enrichment was supposed when NES > 1.50 or < - 1.50 and FDR < 0.25. For comparisons to publically available gene expression data, RNA-sequencing data of BLIMP1^+^ and BLIMP1^-^ Treg cells (GSE62535) was analyzed using RNAExpress (Illumina) and microarray data of CD44^hi^CD62L^lo^ versus CD44^lo^CD62L^hi^ (GSE61077), CD103^-^KLRG1^-^ versus CD103^+^KLRG1^+^ (GSE20366), TIGIT^-^ versus TIGIT^+^ Treg cells (GSE56299) and Tconv versus Treg cells (GSE7460) were analyzed using Geo2R (www.ncbi.nlm.nih.gov/geo/geo2r). The *Id3*^lo^ and *Id3*^hi^ Treg cell RNA-sequencing data reported in this paper are available in the Gene Expression Omnibus database under the accession number GSE75936.

### RT-PCR

Total RNA was isolated using either the RNeasy Kit (Qiagen) or Trizol (Invitrogen). cDNA was prepared with oligo-dT primers using the First Strand cDNA Synthesis Kit (Thermo Scientific), including DNAse I treatment. Quantitative PCR was performed with the Fast Start Universal SYBR Green Master Kit (Roche) on the Mastercycler epgradient S (Eppendorf) with gene-specific primers listed in the Supplemental Experimental Procedures. Hypoxanthine guanine phosphoribosyl transferase 1 (*Hprt1*) transcript levels were used for normalization.

### Statistical analyses

Statistical analyses were performed with two-tailed Student’s t-test, as indicated in the figure legends using Prism 6 software (GraphPad Software, Inc.). All p values < 0.05 were considered significant.

## SUPPLEMENTARY MATERIALS FIGURES AND TABLE


