# Supplementary material for: Regulatory T cells characterized by low Id3 expression are highly suppressive and accumulate during chronic infection
Source: Oncotarget. 2017 Oct 27;8(61):102835–51. doi: 10.18632/oncotarget.22159 (PMC5732693; doi:10.18632/oncotarget.22159)
Supplement: Supplementary file 1 [file oncotarget-08-102835-s001.pdf]

Regulatory T cells characterized by low Id3 expression are highly suppressive and accumulate during chronic infection

SUPPLEMENTARY MATERIALS

REFERENCES

1. Joller N, Lozano E, Burkett PR, Patel B, Xiao S, Zhu C, Xia J, Tan TG, Sefik E, Yajnik V, Sharpe AH, Quintana FJ, Mathis D, et al. Treg cells expressing the coinhibitory molecule TIGIT selectively inhibit proinflammatory Th1 and Th17 cell responses. Immunity. 2014; 40: 569-581.
2. Hill JA, Feuerer M, Tash K, Haxhinasto S, Perez J, Melamed R, Mathis D, Benoist C. Foxp3 transcription-factor-dependent and -independent regulation of the regulatory T cell transcriptional signature. Immunity. 2007; 27: 786-800..
3. Zheng Y, Chaudhry A, Kas A, deRoos P, Kim JM, Chu TT, Corcoran L, Treuting P, Klein U, Rudensky AY. Regulatory T-cell suppressor program co-opts transcription factor IRF4 to control T(H)2 responses. Nature. 2009; 458: 351-356.

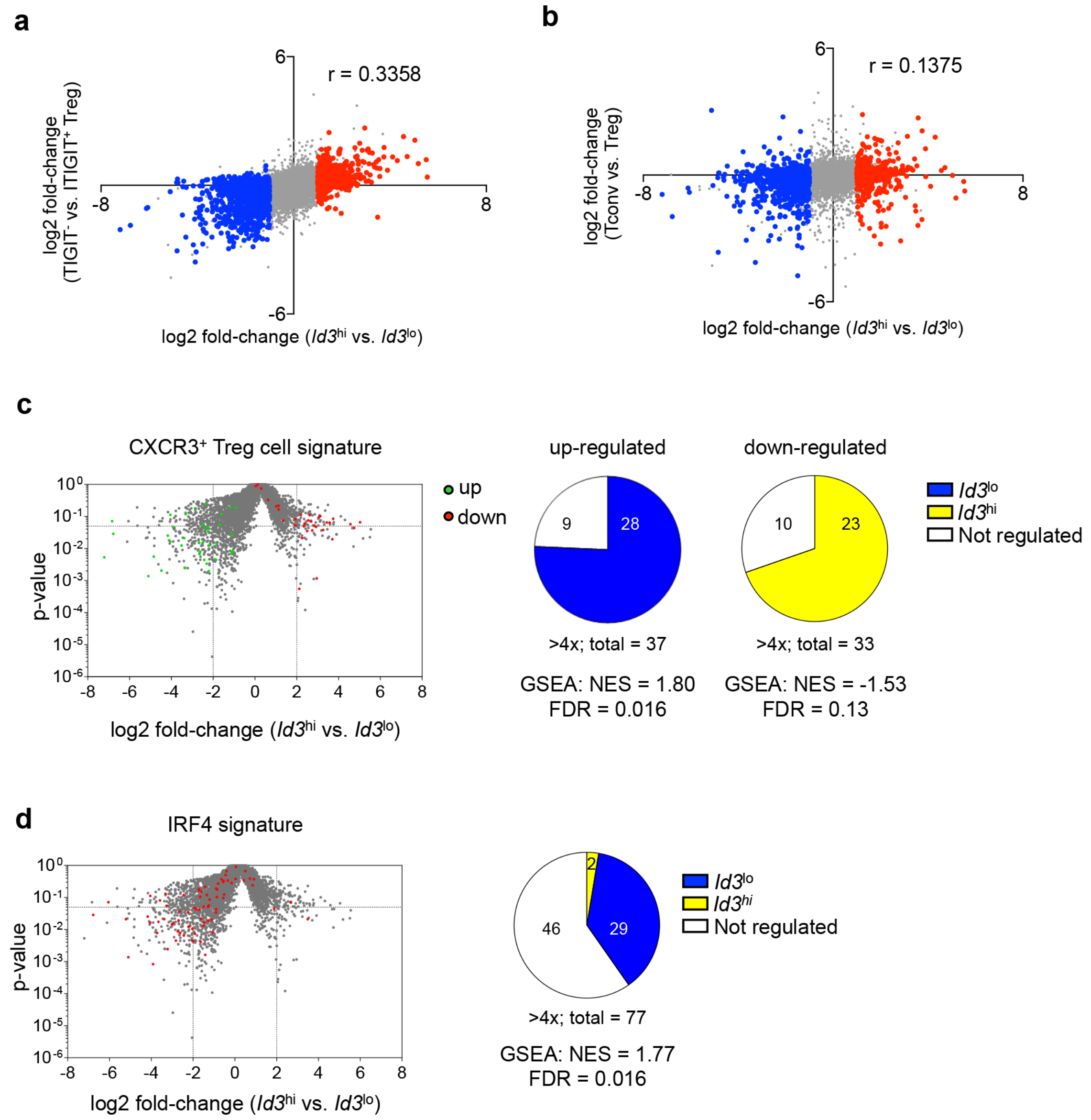

Supplementary Figure 1: *Id3*lo Treg cells express genes upregulated in specialized effector Treg cell subsets. related to Figures 2 and 3 **(a**, **b)** Difference in transcript abundance in *Id3*hi versus *Id3*lo Treg cells plotted against that in TIGIT- versus TIGIT+ Treg cells[1] (a) or in conventional T cells versus Treg cells[2] (b). Colors indicate transcripts significantly up-regulated greater than twofold in *Id3*lo (blue) or *Id3*hi (red) Treg cells. The r-value of Spearman correlation is indicated. **(c**, **d)** Volcano plot comparing the p-value versus log2 fold-change in gene expression of *Id3*hi versus *Id3*lo Treg cells. Genes up- (green) or down-regulated (red) in signatures representing CXCR3+ Treg cells[1] (c) or IRF4 dependent genes[3] (d) are superimposed. Pie charts show the number of respective signature genes up-regulated ≥ 4-fold in *Id3*lo (blue) or *Id3*hi (yellow) Treg cells.

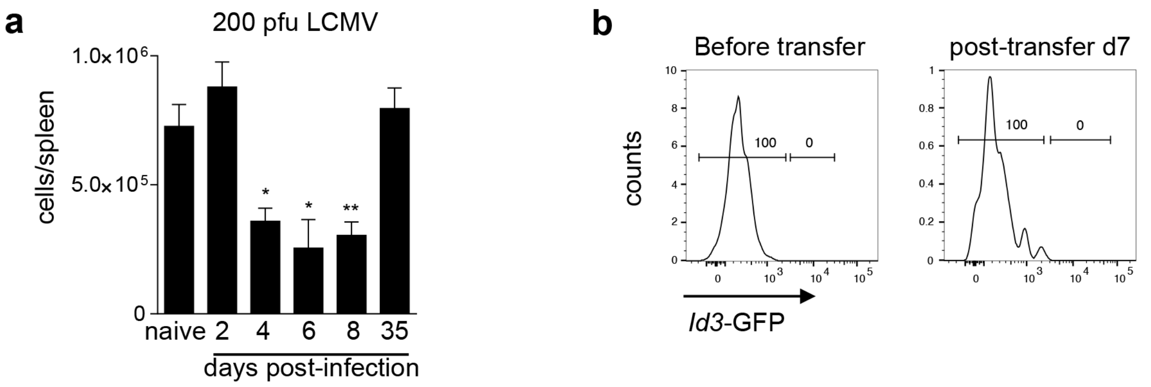

Supplementary Figure 2: *Id3*lo Treg cells do not differentiate into *Id3*hi Treg cells. related to Figure 4 **(a)** Cell number of Treg cells ($CD4^+CD25^+CD45RB^{lo}$) in spleens of naïve or LCMV WE (200 pfu) infected *Id3*$^{GFP/+}$ mice at the indicated time points. Data are represented as mean ± SEM from ≥ 4 individual mice. $^*p<0.05$; $^{**}p < 0.01$; ns = not significant (unpaired Student's t test). **(b)** *Id3*^{lo} Treg cells ($CD4^+CD25^+CD45RB^{lo}$) from naive *Id3*$^{GFP/+}$ mice (Thy1.2) were transferred into wt mice (Thy1.1) and infected with 200 pfu LCMV WE one day after transfer. *Id3* expression of purified *Id3*^{hi} Treg cells immediately before transfer (left) and of transferred cells (Thy1.2$^+$) 7 days post-transfer (right) in spleens of LCMV WE infected mice. Representative of 3 independent experiments.

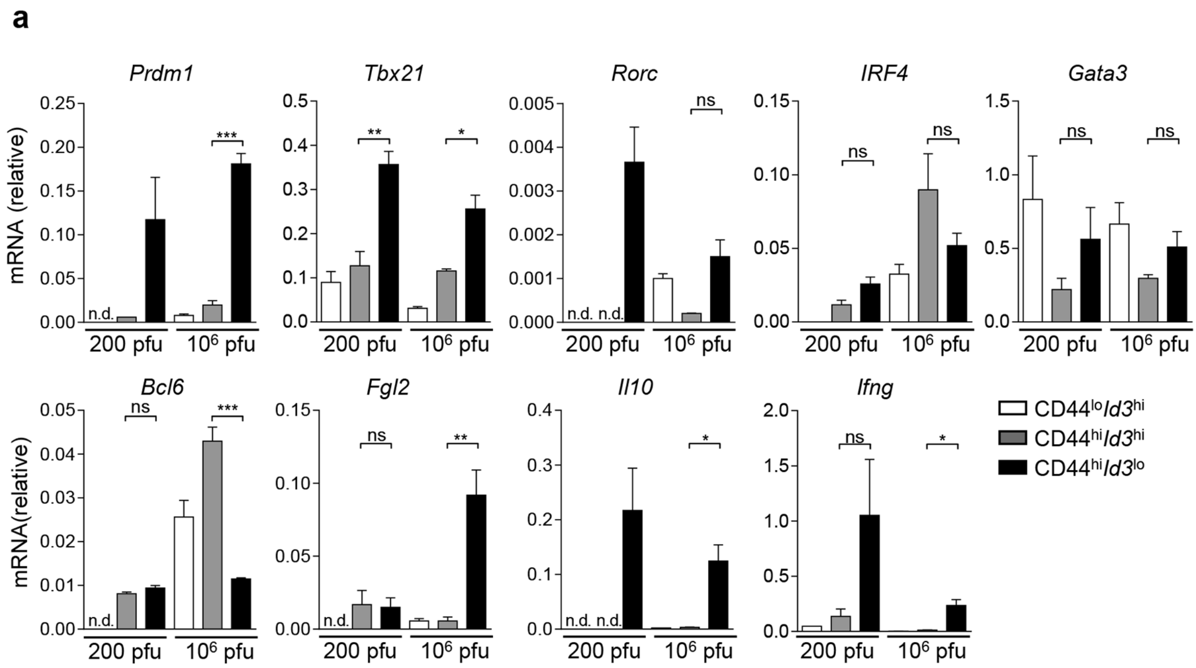

b

	200 Doc			10⁶ Doc		
CD44	lo	hi	hi	lo	hi	hi
Id3	hi	hi	lo	hi	hi	lo
KLRG1	19	235	1860	24	216	2926
PD-1	138	1017	1023	102	687	990
LAG-3	74	138	244	51	154	195
CD103	108	146	350	137	570	727
ICOS	124	837	1484	79	491	1127
CXCR5	137	390	157	138	458	155
CD62L	695	335	290	1049	762	759

Supplementary Figure 3: *Id3*^lo Treg cells have similar phenotype in chronic and acute LCMV infection. related to Figures 6 and 7 **(a)** Quantitative RT-PCR analysis of indicated mRNA transcripts in CD44^{lo}*Id3*^{hi} (white), CD44^{hi}*Id3*^{hi} (grey) or CD44^{hi}*Id3*^{lo} (black) splenic Treg cells (CD4^{+}CD8^{-}CD25^{hi}) from d8 200 pfu or 10^6 pfu LCMV Docile infected *Id3*$^{GFP/+}$ mice. Data are represented as mean ± SEM from 3 individual mice. n.d. = not detectable; $^{*}p < 0.05$; $^{**}p < 0.01$; $^{***}<0.001$; ns = not significant (unpaired Student's t test) **(b)** Flow cytometric protein level analysis of indicated surface markers on CD44^{lo}*Id3*^{hi}, CD44^{hi}*Id3*^{hi}, CD44^{hi}*Id3*^{lo} Treg cells (CD4^{+}CD25^{hi}) of d8 200 pfu or 10^6 pfu LCMV Docile infected *Id3*$^{GFP/+}$ mice. Numbers are the mean fluorescence intensity of the indicated surface proteins from 3 individual mice.

Supplementary Table 1: RT-PCR primer sequences

Gene	forward	reverse
Hprt	GTTAAGCAGTACAGCCCCAAA	AGGGCATATCCAACAACAAACTT
Prdm1	AAGAGGTTATTGGCGTGGTAAG	TAGACTTCACCGATGAGGGGT
Ifng	TCAGCAACAACATAAGCGTC	GGTTGTTGACCTCAAACTTGG
Fgl2	ACATTGAGAACTACGTGGACAAC	ACACTTGGAACACTTGCCATC
Irf4	TCCGACAGTGGTTGATCGAC	CCTCACGATTGTAGTCCTGCTT
Bcl6	CCGGCACGCTAGTGATGTT	TGTCTTATGGGCTCTAAACTGCT
Rorc	AAGATCTGCAGCTTTTCCACA	TTTGGAACTGGCTTTCCATC
Gata3	CTCGGCCATTCGTACATGGAA	GGATACCTCTGCACCGTAGC
Il10	GAAGACCCTCAGGATGCGG	CCTGCTCCACTGCCTTGCT
